# TRF2 Protein Interacts with Core Histones to Stabilize Chromosome Ends*

**DOI:** 10.1074/jbc.M116.719021

**Published:** 2016-08-11

**Authors:** Akimitsu Konishi, Takashi Izumi, Shigeomi Shimizu

**Affiliations:** From the ‡ Department of Pathological Cell Biology and; § Medical Top Track Program, Medical Research Institute, Tokyo Medical and Dental University, Yushima, Bunkyo-ku, Tokyo 113-8510, Japan,; the ¶ Department of Biochemistry, Gunma University Graduate School of Medicine, 3-39-22 Showa, Maebashi, Gunma 371-8511, Japan, and; the ‖ Laboratory of Cell Biology and Genetics, The Rockefeller University, New York, New York 10065

**Keywords:** chromatin, chromosomes, DNA damage response, histone, telomere, GAR domain, core histone

## Abstract

Mammalian chromosome ends are protected by a specialized nucleoprotein complex called telomeres. Both shelterin, a telomere-specific multi-protein complex, and higher order telomeric chromatin structures combine to stabilize the chromosome ends. Here, we showed that TRF2, a component of shelterin, binds to core histones to protect chromosome ends from inappropriate DNA damage response and loss of telomeric DNA. The N-terminal Gly/Arg-rich domain (GAR domain) of TRF2 directly binds to the globular domain of core histones. The conserved arginine residues in the GAR domain of TRF2 are required for this interaction. A TRF2 mutant with these arginine residues substituted by alanine lost the ability to protect telomeres and induced rapid telomere shortening caused by the cleavage of a loop structure of the telomeric chromatin. These findings showed a previously unnoticed interaction between the shelterin complex and nucleosomal histones to stabilize the chromosome ends.

## Introduction

The ends of eukaryotic chromosomes are stabilized by specialized structures called telomeres. Failure of the proper function of telomeres leads to a DNA damage response and inappropriate DNA repair at chromosome ends (1). Mammalian telomeres comprise a long array of tandem TTAGGG repeat DNA and shelterin, a telomere-specific protein complex (2). Electron microscopy analysis revealed that telomeres are organized in a large lasso-like structure called a t-loop (3, 4). Recently, t-loops were also detected by a super-resolution fluorescence imaging method (5). t-loops are believed to be formed through strand invasion of duplex telomeric repeat by the 3′ overhang, and their formation requires both homologous recombination (HR)^2^ and shelterin components (6). It has been proposed that t-loops protect the telomere terminus from DNA damage response and the DNA repair machinery.

Telomeric nucleosomes have a similar composition to non-telomeric nucleosomes: they contain core histones H2A, H2B, H3, H4, and linker histone H1 (4). However, the higher order structure of telomeric chromatin is different from bulk nuclear chromatin. Telomeric nucleosomes have shorter repeat sizes than bulk nucleosomes and are hypersensitive to micrococcal nuclease (7, 8). Reconstituted nucleosomes on TTAGGG repeats show higher mobility than on other sequences (9). Moreover, telomeric chromatin is enriched for heterochromatin modification, such as tri-methylation of H3K9 and H4K20, and loss of these marks affects telomere length regulation (10).

Telomere function is critically dependent on the shelterin complex. TRF2 is a component of the shelterin complex that localizes at telomeres to protect chromosome ends from inappropriate DNA damage response and the DNA repair machinery (11). Deletion of TRF2 leads to ataxia telangiectasia-mutated (ATM)-dependent DNA damage response at chromosome ends, detected as the occurrence of telomere dysfunction-induced foci (TIFs), which results in end-to-end telomere fusions mediated by non-homologous end joining (NHEJ) (12, 13). TRF2 consists of three domains, the TRF homology (TRFH) domain, a C-terminal Myb/SANT DNA-binding domain, and the Gly/Arg-rich domain (GAR domain; previously referred to as the basic domain).

The GAR domain, which is located at the N terminus of TRF2, is rich in Gly/Arg residues and highly basic, and is important to stabilize the chromosome ends by repressing cleavage of the t-loop. Ectopic expression of TRF2 lacking the GAR domain (TRF2ΔB) induces stochastic telomere deletions and accumulation of circular telomeric DNA (t-circle), as expected from excision of the t-loop mediated by the HR machinery (14). Recent biochemical analyses showed that the GAR domain binds to a Holliday junction, suggesting that the GAR domain prevents t-loop cleavage by physically blocking the junction structure (15, 16). Another group has reported that the GAR domain of TRF2, a telomere-repeat-encoding RNA (TERRA), and the origin recognition complex (ORC) form a ternary complex to stabilize the t-loop (17, 18). However, the molecular mechanism of the regulation of telomere function by the GAR domain of TRF2 is largely unknown.

In the present study, we report that the GAR domain of TRF2 directly binds to core histones and that this interaction is required to stabilize the chromosome ends.

## Results

### 

#### 

##### TRF2 Binds to Core Histones through the GAR Domain

**FIGURE 1.**
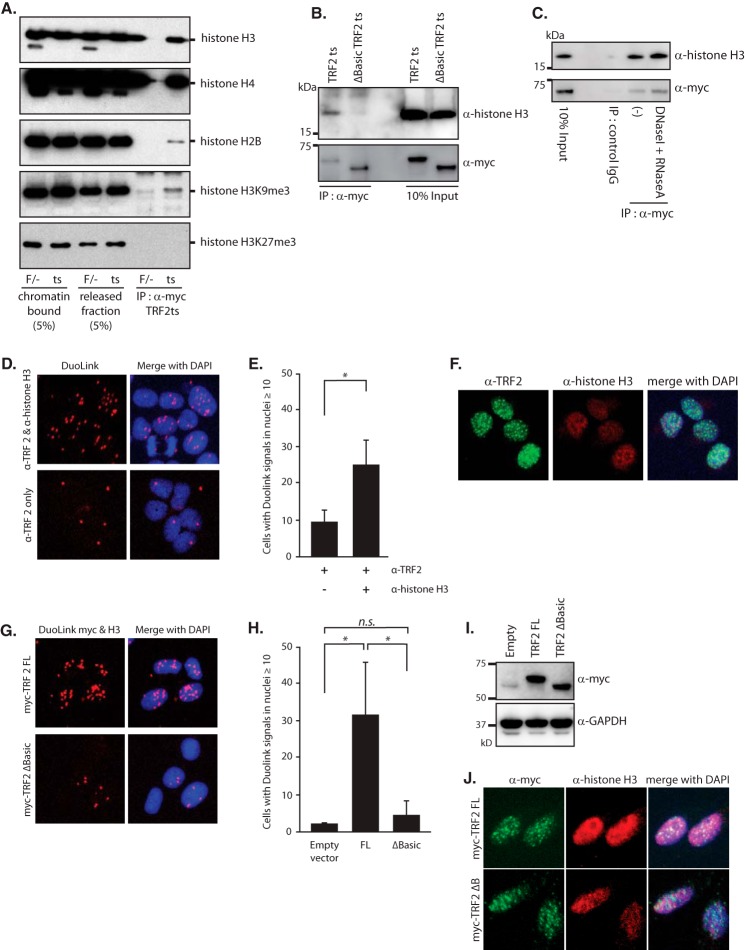
**Interaction between TRF2 and core histones.** *A*, co-immunoprecipitation of TRF2 and core histones. Chromatin-bound and -released fractions were obtained from Myc-tagged TRF2ts-expressing cells (*ts*) and TRF2^F/−^ (*F*/−) parental cells. Released fractions were subjected to co-immunoprecipitation with anti-Myc antibody. Immunoprecipitates were then subjected to immunoblotting with the indicated antibodies. *B*, co-immunoprecipitation of ΔBasic TRF2 and core histones. The chromatin-released fraction from Myc-tagged TRF2ts-expressing cells and Myc-tagged ΔBasic TRF2ts-expressing cells were subjected to co-immunoprecipitation with anti-Myc antibody. Immunoprecipitates were then subjected to immunoblotting with anti-histone H3 and anti-Myc antibodies. *C*, co-immunoprecipitation of TRF2 and core histones after removal of DNA and RNA. The chromatin-released fraction from Myc-tagged TRF2ts-expressing cells was treated with DNase I and RNase A, and then subjected to co-immunoprecipitation with anti-Myc antibody. Immunoprecipitates were then subjected to immunoblotting with anti-histone H3 and anti-Myc antibodies. Immunoprecipitation using control IgG served as a negative control. *D*, Duolink PLA assay of TRF2 and histone H3. The Duolink assay was performed using anti-TRF2 and anti-histone H3 antibodies in HeLa cells. Duolink signals are visualized in red merged with DAPI staining (*blue*). Duolink using only anti-TRF2 antibody served as a negative control. *E*, quantification of the Duolink assay in *D*. Bars represent mean values from three independent experiments ± S.D. (*error bars*). *, *p* < 0.05, based on unpaired Student's *t* test. *F*, regular immunofluorescence images for TRF2 and histone H3. The merged image is shown with DNA staining using DAPI. *G*, Duolink PLA assay of TRF2 ΔBasic and histone H3. HeLa cells were transformed to express Myc-tagged full-length TRF2 (*TRF2 FL*) or TRF2 ΔBasic. Cells were then subjected to Duolink PLA assay using anti-Myc and anti-histone H3 antibodies. Duolink signals are visualized in red merged with DAPI staining (*blue*). *H*, quantification of Duolink assay in *G*. Bars represent mean values from three independent experiments ± S.D. (*error bars*). *, *p* < 0.05, *n.s.*, not significant, based on one-way ANOVA with Tukey's test. *I*, immunoblotting to verify the equal expression of Myc-tagged FL, ΔB TRF2 in HeLa cells. GAPDH served as a loading control. *J*, regular immunofluorescence images using anti-Myc and anti-histone H3 antibodies to verify equal localization of Myc-TRF2 FL and Myc-TRF2 ΔB. The merged image is shown with DNA staining using DAPI.

In the course of identifying the shelterin complex-associated proteins, we noticed that core histones were bound to TRF2. As shown previously, the whole shelterin complex can be isolated from the chromatin under mild conditions in cells expressing the temperature-sensitive TRF2 mutant (TRF2ts) (19). TRF2ts is released from the isolated chromatin together with other shelterin components by a temperature shift *in vitro*. Co-immunoprecipitation analysis using the released fraction from the isolated chromatin of TRF2ts cells showed that core histones were co-immunoprecipitated with TRF2ts protein. Histone H3, H4, and H2B were confirmed to be precipitated together with TRF2ts by immunoblotting (Fig. 1*A*). Telomeric chromatin is enriched for tri-methylated H3 lysine 9 (H3K9), but not for tri-methylated H3 lysine 27 (H3K27) (20). Likewise, TRF2ts was bound to tri-methylated H3K9, but not to tri-methylated H3K27 (Fig. 1*A*), suggesting that TRF2ts was bound to core histone localized at the telomeric chromatin. This interaction was largely diminished in N-terminal GAR domain-deleted TRF2ts mutant (ΔBasic TRF2ts) (Fig. 1*B*), suggesting that the interaction between TRF2 and the core histones was dependent on the GAR domain of TRF2.

It has been reported that the GAR domain of TRF2 binds to DNA or RNA (15, 18). To exclude the possibility that TRF2 GAR domain binds to core histones through the nucleic acid, we hydrolyzed DNA and RNA in the released fraction by DNase I and RNase A. Even after the DNase I and RNase A treatment, core histones were still co-immunoprecipitated with TRF2ts protein (Fig. 1*C*), suggesting that the binding between the GAR domain of TRF2 and core histones was not dependent on the nucleic acid.

To corroborate the interaction between TRF2 and core histones, we used the Duolink *in situ* proximity ligation assay (PLA) method, which gives a fluorescent single signal if two different antibody epitopes are localized in close proximity (within 40 nm) (21). Using antibodies toward TRF2 and histone H3, we could detect more than 10 positive Duolink signals in 26% of HeLa cells (Fig. 1, *D* and *E*). Under these conditions, the different distribution of TRF2 and histone H3 was confirmed by regular immunofluorescence. TRF2 was stained at telomeres as a dotted pattern and histone H3 was stained throughout the nuclei, as expected (Fig. 1*F*). HeLa cells expressing exogenous Myc-tagged full-length TRF2 (Myc-TRF2 FL) showed positive Duolink signals using anti-Myc and anti-histone H3 antibodies at a similar rate. However, the Duolink signal was largely diminished in cells expressing the GAR domain-deleted TRF2 (Myc-TRF2 ΔBasic) (Fig. 1, *G* and *H*). As reported previously (14), the TRF2ΔBasic protein was localized at telomeres similarly to TRF2FL (Fig. 1, *I* and *J*). These results further indicated that TRF2 was bound to core histones through the GAR domain.

##### Direct Binding of TRF2 GAR Domain to Core Histones

**FIGURE 2.**
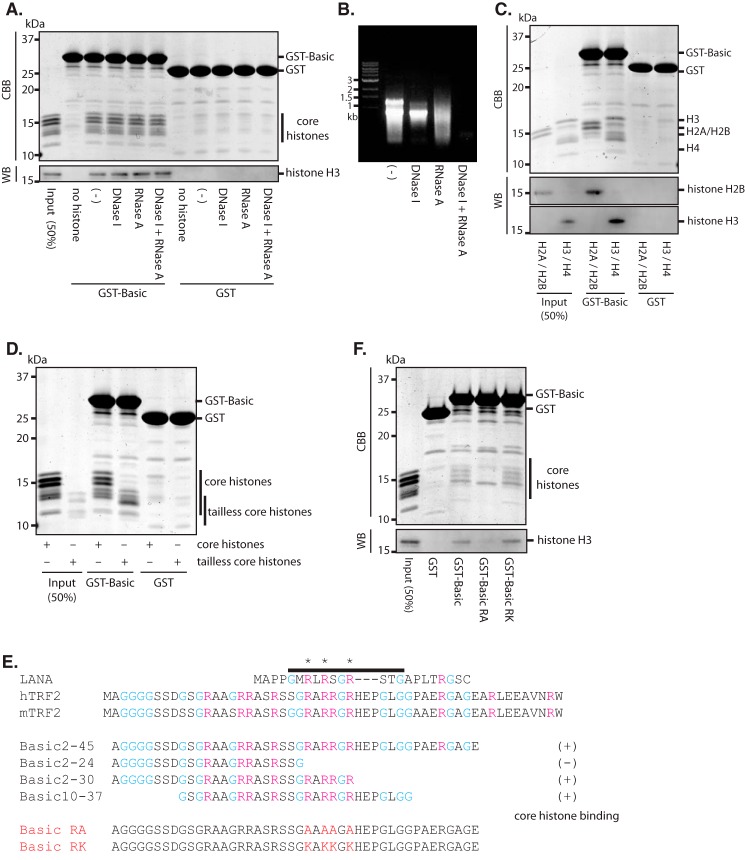
**Direct binding of the GAR domain of TRF2 and core histones.** *A*, *in vitro* binding assay for the GAR domain of TRF2 and core histones. Recombinant GST-fused TRF2 GAR domain (*GST-Basic*) and GST protein (*GST*) were treated with or without DNase I, RNase A, or both DNase I and RNase A, and then captured by glutathione-conjugated beads and incubated with core histones purified from HeLa cells. Beads were washed extensively and then subjected to SDS-PAGE. The *upper panel* shows CBB staining of an SDS-PAGE gel. The *bottom panel* shows immunoblotting using an anti-histone H3 antibody. *WB*, Western blot. *B*, complete hydrolysis of the nucleic acid contaminants in the bacterial lysate used for *in vitro* binding assay in *A*. Nucleic acid extracted from the lysate was subjected to the agarose gel electrophoresis. The gel was stained with ethidium bromide. *C*, *in vitro* binding assay for histone H2A-H2B and H3-H4. GST-Basic protein treated with DNase I and RNase A was captured by glutathione-conjugated beads. Beads were incubated with purified H2A-H2B dimer or H3-H4 tetramer and then subjected to SDS-PAGE after the extensive wash. The *top panel* shows CBB staining of an SDS-PAGE gel. The *middle* and *bottom panels* show immunoblotting for histone H2B and histone H3, respectively. *D*, *in vitro* binding assay for tailless core histones. DNase I- and RNase A-treated GST-Basic protein was captured by glutathione-conjugated beads. Beads were incubated with purified core histones or tailless core histones and then subjected to SDS-PAGE after the extensive wash, followed by CBB staining. *E*, multiple sequence alignments of the basic domain from LANA, human TRF2 (hTRF2), and mouse TRF2 (mTRF2). Amino acid residues required for LANA-histone binding are marked by the *line*. Conserved arginine residues are marked by *asterisks*. The result of an *in vitro* binding assay for GST-Basic deletion mutants and core histones is shown in the middle. The residues that were changed to alanine or lysine are marked in *red* (*Basic RA* and *Basic RK*). *F*, *in vitro* binding assay for GST-Basic mutants and core histones. GST-fused Basic, GST-Basic RA, and GST-Basic RK proteins treated with DNase I and RNase A were captured by glutathione-conjugated beads. Beads were incubated with purified core histones and then subjected to SDS-PAGE after the extensive wash. The *top panel* shows CBB staining of an SDS-PAGE gel, and the *bottom panel* shows immunoblotting for histone H3.

We then investigated whether the TRF2 GAR domain binds to core histone directly. Bacteria expressing recombinant GST-fused GAR domain (GST-Basic) were incubated with purified core histones and then pulled down by glutathione-conjugated beads. As a result, all four core histones, H2A, H2B, H3, and H4, were pulled down with GST-Basic (Fig. 2*A*). To exclude the possibility that GST-Basic binds to core histones through the nucleic acid, we treated recombinant GST-Basic protein and core histones with DNase I, RNase A, or both. Neither DNase I nor RNase A treatment affected the affinity between GST-Basic and the core histones (Fig. 2*A*), although the nucleic acid contaminants were completely hydrolyzed by DNase I and RNase A (Fig. 2*B*). We then added DNase I and RNase A treatment to further *in vitro* binding assays.

The nucleosome core is formed by two H2A/H2B dimers and a H3/H4 tetramer. Therefore, we asked which component of the core histones interacts with the GAR domain of TRF2. The GST-Basic protein was incubated with H2A/H2B and H3/H4 separately and bound to both H2A/H2B and H3/H4 (Fig. 2*C*). Structurally, core histones can be separated to the globular domain and N and C terminus tail region. We also asked whether the globular domain or the tail region binds to the GAR domain of TRF2. The GST-Basic protein was incubated with core histones and the tailless core histones, which were generated by trypsin partial digestion of nucleosomes, followed by histone purification. GST-Basic could bind to the tailless core histones (Fig. 2*D*), suggesting that the histone tail region is not required for the interaction between TRF2 and core histones.

We then identified the residues in the GAR domain of TRF2 that are required to bind to the core histones. It has been reported that Kaposi sarcoma-associated herpesvirus latency-associated nuclear antigen (LANA), which has an N-terminal basic domain enriched with basic amino acid residues, like TRF2, binds to the globular region of core histone H2A/H2B dimer through its basic domain. LANA residues 5–15 are required for histone binding. X-ray crystallography showed that arginine residues 7, 9, and 12 bind to the acidic groove in the globular domain of H2A/H2B, and these arginine residues are required for the interaction (22). A similar amino acid sequence to that of LANA 5–15 was found in the GAR domain of TRF2, and all three essential arginine residues for histone binding of LANA are conserved in vertebrates TRF2 (Fig. 2*E*). There is an additional arginine residue in the GAR domain of TRF2, and this arginine (residue 28) is conserved in vertebrate TRF2 (Fig. 2*E*). We hypothesized that these four conserved arginine residues in the GAR domain of TRF2 form the interaction between TRF2 and core histones, like LANA. We tested this hypothesis by an *in vitro* binding assay. Truncated GST-Basic2–30 or GST-Basic10–37, which both contain these four arginines, could bind to the core histones, whereas the GST-Basic2–24, which lacks the four arginines, did not bind to the core histones (Fig. 2*E*). Indeed, GST-Basic in which all four arginine residues were substituted by alanine residues (GST-Basic RA) completely lost the ability to bind to the core histones (Fig. 2, *E* and *F*).

Taken together, these results indicated that the GAR domain of TRF2 binds directly to the globular domain of core histones, both H3/H4 and H2A/H2B, and the four conserved arginine residues in the GAR domain of TRF2 are required for binding to the core histones.

##### TRF2 GAR Domain Binds to Core Histones in Vivo

**FIGURE 3.**
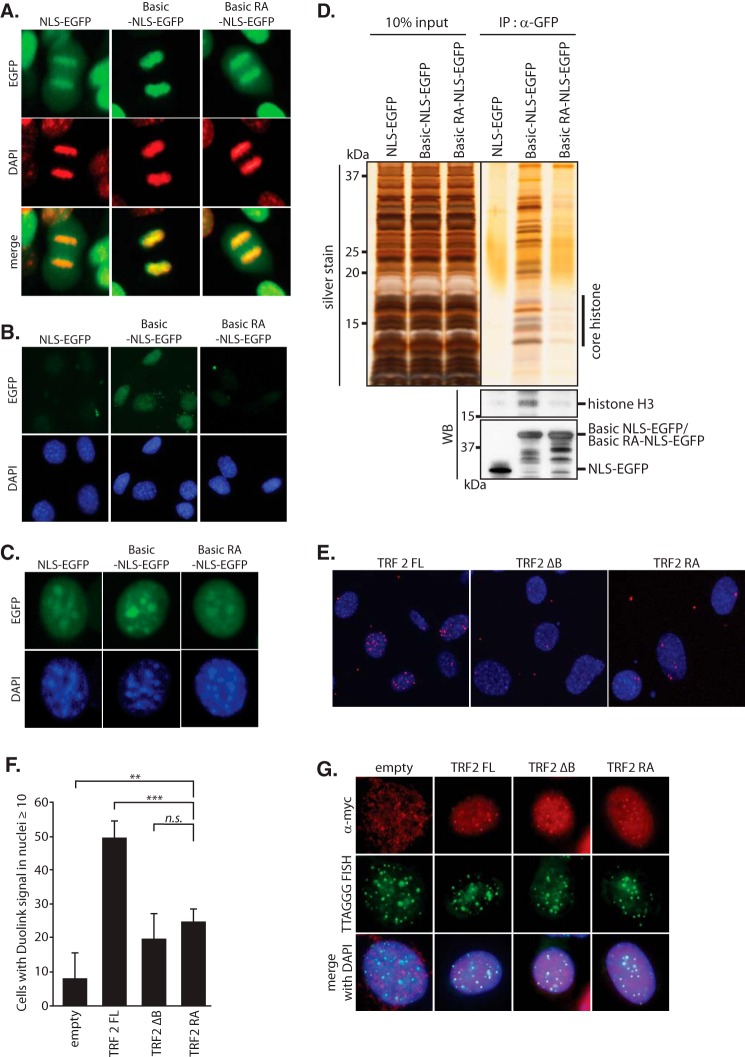
**Interaction between the GAR domain of TRF2 and core histones *in vivo*.** *A*, chromatin bound Basic-NLS-EGFP in metaphase. NLS-EGFP control, Basic-NLS-EGFP, and Basic-RA-NLS-EGFP were introduced into NIH-3T3 cells. Images of metaphase cells were captured with a fluorescence microscope. The *top panel* shows the EGFP signal, the *middle panel* shows DAPI staining pseudo-colored in red, and the *bottom panel* shows the merged image. *B*, nuclear localization of Basic-NLS-EGFP after nucleoplasmic pre-extraction. NIH-3T3 cells expressing NLS-EGFP, Basic-NLS-EGFP, and Basic-RA-NLS-EGFP were extracted *in situ* with the nucleoplasm removal buffer. The images of DAPI staining and EGFP signal were captured with a fluorescence microscope. *C*, fluorescence microscopic images of Basic-NLS-EGFP, Basic-RA-NLS-EGFP, and NLS-EGFP to verify the equal nuclear localization in interphase NIH-3T3 cell before nucleoplasmic pre-extraction. *D*, co-immunoprecipitation of Basic-NLS-EGFP and core histones. NIH-3T3 cells expressing NLS-EGFP, Basic-NLS-EGFP, and Basic-RA-NLS-EGFP were extracted with radioimmunoprecipitation assay buffer. Cell extracts were subjected to co-immunoprecipitation with an anti-GFP antibody. Immunoprecipitants were then applied to SDS-PAGE. Silver staining (*top*) and immunoblotting for histone H3 (*middle*) and EGFP (*bottom*) are presented. *WB*, Western blot. *E*, Duolink PLA assay of TRF2 mutants and histone H3. Myc-tagged TRF2 FL-, ΔB-, and RA-expressing MEFs were subjected to Duolink PLA assay using anti-Myc and anti-histone H3 antibodies. Duolink signals are visualized in red merged with DAPI staining (*blue*). *F*, quantification of Duolink assay in *E*. Bars represent mean values from three independent experiments ± S.D. (*error bars*). **, *p* < 0.01, ***, *p* < 0.001, *n.s.*, not significant, based on one-way ANOVA with Dunnett's test. *G*, IF-FISH images to verify the equal telomere localization of TRF2 FL, ΔB, and RA. Myc-tagged TRF2 FL-, ΔB-, and RA-expressing MEFs were subjected to IF-FISH. Immunofluorescence for Myc (*red*), FISH using telomeric TTAGGG probe (*green*), and merged images with DAPI (*blue*) are presented.

We next investigated whether the GAR domain of TRF2 binds to core histones *in vivo*. First, we studied the localization of the GAR domain fused with the nuclear localization signal (NLS) and enhanced green fluorescent protein (EGFP) at its C terminus (Basic-NLS-EGFP). During metaphase, the Basic-NLS-EGFP signal completely overlapped with the chromatin, whereas the NLS-EGFP control was released from the chromatin and spread into the cytoplasm (Fig. 3*A*). In interphase cells, both NLS-EGFP and Basic-NLS-EGFP were localized in nuclei (Fig. 3*C*); however, NLS-EGFP was completely removed from the nuclei by pre-extraction of the nucleoplasmic fraction before fixing the cells (Fig. 3*B*). By contrast, Basic-NLS-EGFP was localized in nuclei even after pre-extraction (Fig. 3*B*). Indeed, core histones could be co-immunoprecipitated with Basic-NLS-EGFP (Fig. 3*D*). These results indicated that the TRF2 basic domain binds to the core histones *in vivo*.

We then asked whether the conserved arginine residues in the GAR domain are also important for histone binding *in vivo*. Basic-RA-NLS-EGFP in which the four arginines are substituted by alanines behaved like the NLS-EGFP control. Basic-RA-NLS-EGFP was released from chromatin in metaphase cells (Fig. 3*A*) and was completely removed by pre-extraction in interphase cells (Fig. 3*B*). Co-immunoprecipitation of core histones with anti-GFP antibody in Basic-RA-NLS-EGFP-expressing cells was largely diminished (Fig. 3*D*). Moreover, TRF2 with alanine substitutions of four arginines in the GAR domain (TRF2 RA) showed decreased binding to histone H3 in mouse embryonic fibroblast cells, as assessed by Duolink PLA assay (Fig. 3, *E–G*).

Taken together, these results showed that the GAR domain of TRF2 binds to core histones *in vivo*, and that the four arginine residues in the GAR domain are important for this interaction.

##### Interaction between TRF2 and Core Histones Is Required for Chromosome End Protection from DNA Damage Response

**FIGURE 4.**
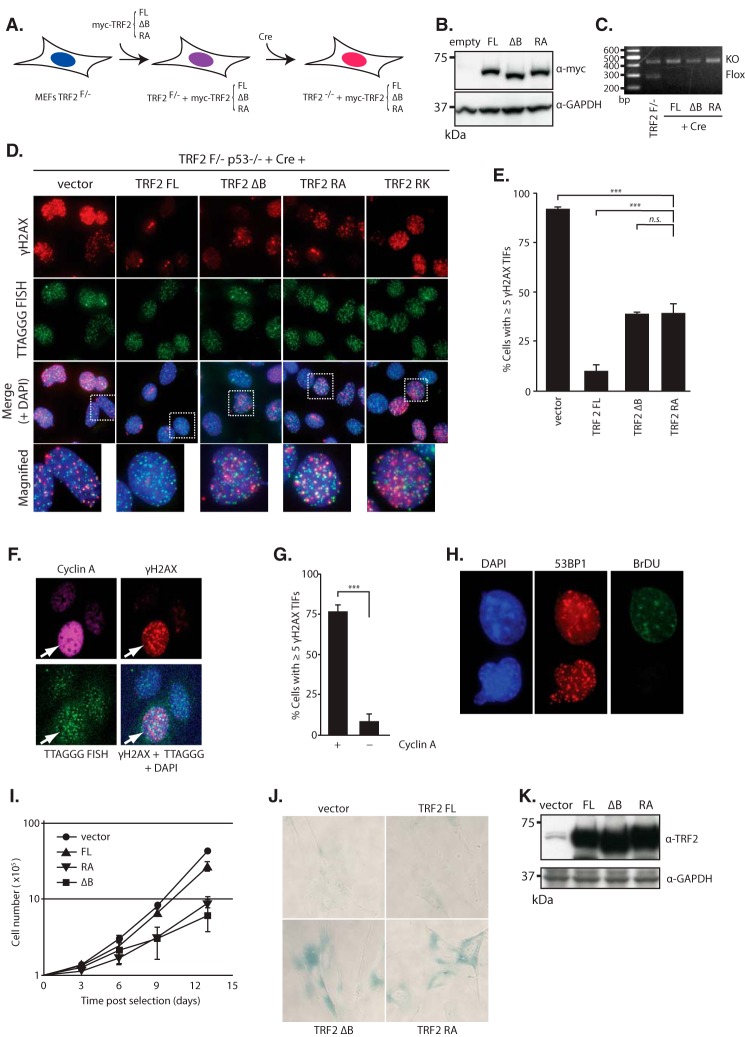
**Loss of telomere protection in TRF2-histone interaction-deficient cells.** *A*, schematic of the experiment for TRF2 complementation in MEFs with TRF2 mutant alleles. TRF2^F/−^ MEFs were retrovirally introduced with a wild-type TRF2 (*FL*) or mutant (Δ*B* and *RA*) alleles and then were induced with Cre-mediated deletion of TRF2 flox allele. *B*, immunoblotting to verify the equal expression of Myc-tagged FL, ΔB, and RA mutated TRF2. GAPDH served as a loading control. *C*, PCR to verify Cre-mediated deletion of the endogenous allele in TRF2 FL-, ΔB-, and RA-complemented MEFs. *D*, TIF induction in TRF2 RA cells. TRF2 FL-, ΔB-, RA-, and RK-complemented MEFs were subjected to a TIF assay. Immunofluorescence for γH2AX (*red*), FISH using telomeric TTAGGG probe (*green*), and merged images with DAPI (*blue*) are presented. The *white dotted boxes* indicate the site of the magnified images. *E*, quantification of the TIF assay in *D*. Bars represent mean values from three independent experiments ± S.D. (*error bars*). ***, *p* < 0.001, *n.s.*, not significant, based on one-way ANOVA with Dunnett's test. *F*, cell cycle-specific induction of TIF in TRF2 RA cells. TRF2 RA mutant-complemented MEFs were co-immunostained for cyclin A (*magenta*) and γH2AX (*red*), and FISH detection with telomeric TTAGGG probe (*green*) was performed. An *arrow* indicates a cyclin A-positive cell. *G*, quantification of the TIF assay in *F*. Bars represent mean values from three independent experiments ± S.D. (*error bars*). ***, *p* < 0.001, based on unpaired Student's *t* test. *H*, immunofluorescence image for BrdU (*green*) and 53BP1 (*red*) of TRF2 RA-complemented MEFs. DNA was visualized with DAPI (*blue*). *I*, proliferation of IMR90 cells expressing TRF2 FL, TRF2 RA, and TRF2 ΔB. Cells were retrovirally transfected with the indicated cDNA. The growth curves after drug selection are presented. Data represent mean values from three independent experiments ± S.D. (*error bars*). *J*, SA-β-gal assay of IMR90 cells overexpressing TRF2 FL, TRF2 RA, and TRF2 ΔB at day 10 after drug selection. *K*, immunoblotting to verify the equal expression of TRF2 FL, TRF2 RA, and TRF2 ΔB in IMR90 cells. GAPDH served as a loading control.

We investigated the role of the interaction between TRF2 and core histones. The GAR domain of TRF2 has been reported to be required to protect chromosome ends (14). TRF2^flox/−^ mouse embryonic fibroblast cells expressing Cre recombinase to delete the TRF2 allele lost their ability to protect chromosome ends such that they showed TIFs (12) (Fig. 4, *A–D*). The formation of TIFs was completely inhibited by expression of the wild-type full-length TRF2 allele (TRF2 FL) by retroviral introduction before Cre recombination (Fig. 4, *D* and *E*). Introduction of a TRF2 allele lacking the GAR (TRF2 ΔB) could not rescue the TIF formation completely, as reported previously (14). TIFs were still formed in ∼40% of cells expressing TRF2 ΔB (Fig. 4, *D* and *E*). The TRF2 RA allele, which lost the core histone binding ability, also could not rescue the TIF formation completely. The cells expressing TRF2 RA formed TIFs at a similar rate to those expressing TRF2ΔB (Fig. 4, *D* and *E*). About 75% of TIF-positive cells expressing TRF2 RA could be stained with cyclin A (Fig. 4, *F* and *G*). BrdU labeling did not match TIF-positive cells (Fig. 4*H*), suggesting that loss of telomere protection in TRF2 RA-expressing cells occurred in the S/G_2_ cell cycle phase.

We then investigated the consequence of the loss of the interaction of TRF2 with the core histones. Overexpression of TRF2 ΔB resulted in premature senescence in primary human fibroblasts (14). We asked whether loss of the histone interaction of TRF2 also induced premature senescence. The proliferation of IMR90 human fibroblast cells expressing TRF2 RA was largely diminished, similar to TRF2 ΔB, and the cells acquired a flattened shape and became positive for senescence-associated β-galactosidase (SA-β-gal), indicating senescent morphology (Fig. 4, *I–K*).

Taken together, these data indicated that the interaction between TRF2 and the core histone is important for chromosome end protection in the S/G_2_ cell cycle phase, and loss of the interaction inhibits cell proliferation and leads to premature senescence.

##### Interaction between TRF2 and Core Histones Is Required for Telomeric Loop Stabilization

**FIGURE 5.**
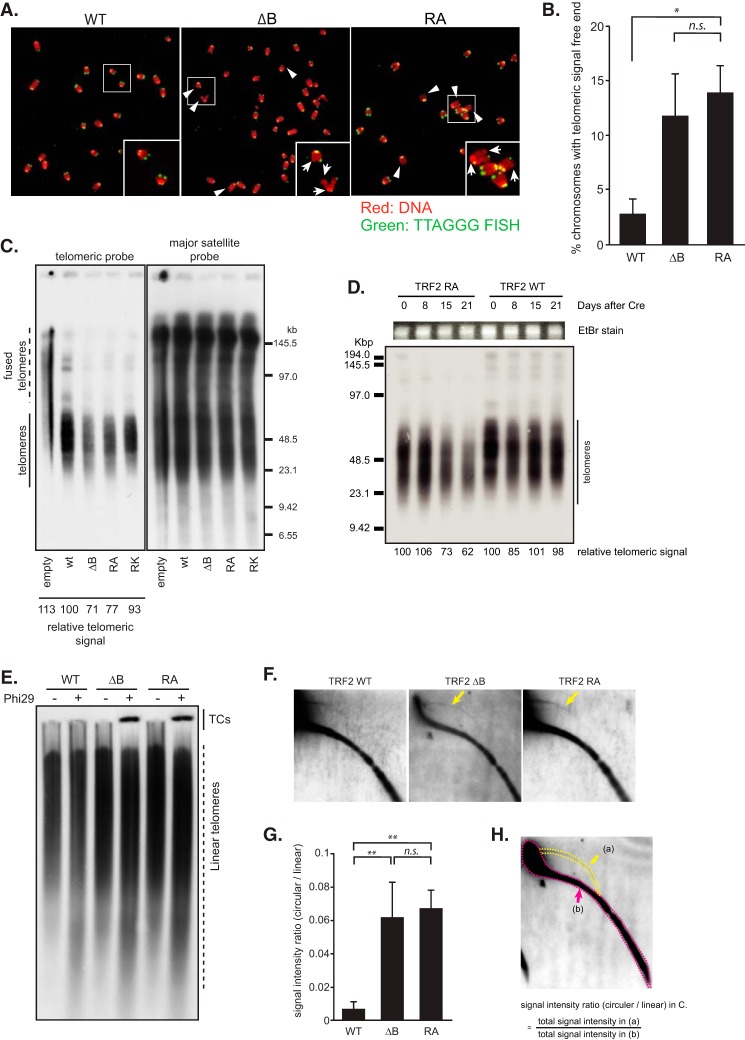
**Rapid telomere DNA loss and t-circle generation by loss of histone binding of TRF2.** *A*, metaphase FISH analysis using telomeric TTAGGG probe (*green*) in TRF2 FL, TRF2 RA, and TRF2 ΔB cells. DNA was visualized with DAPI as a pseudo-colored *red. Arrows* and *arrowheads* indicate chromosomes with telomeric signal free ends. The *white boxes* indicate the sites of the magnified images (*bottom right*). *B*, quantification of metaphase FISH analysis in *A*. Bars represent mean values from three independent experiments ± S.D. (*error bars*). *, *p* < 0.05, *n.s.*, not significant, based on one-way ANOVA with Dunnett's test. *C*, loss of telomere signals in TRF2 mutants. TRF2^F/−^ MEFs expressing TRF2 wt, ΔB, and RA mutant were harvested 14 days after Cre treatment and subjected to Southern blotting of terminal restriction fragments using a DIG-labeled telomeric TTAGGG probe (*left*). The *numbers* below the lane represent the relative telomeric signal normalized by the major satellite probe signal (*right*). *D*, rapid telomeric DNA loss in TRF2 RA-complemented MEFs. The cells were harvested at the indicated time point after Cre treatment and subjected to Southern blotting using a DIG-labeled telomeric TTAGGG probe (*bottom*). DNA visualized with EtBr (*top*) served as a loading control. *E*, t-circle production in TRF2 RA-complemented MEFs detected by t-circle amplification assay. Phi29-dependent t-circles (*TCs*) were detected 4 days after Cre treatment of TRF2^F/−^ MEFs expressing wt-, ΔB-, and RA-mutated TRF2 cDNA. *F*, t-circle production in TRF2 RA-complemented MEFs detected by 2D Southern blotting. Cells were harvested 5 days after Cre treatment. Equal amounts of MboI-digested genomic DNA were subjected neutral 2D agarose electrophoresis. Telomeric DNA was visualized by Southern blotting using a DIG-labeled TTAGGG probe. *Arrows* indicate t-circles. *G*, quantification of 2D Southern blotting in *F*. Bars represent mean values from three independent experiments ± S.D. (*error bars*). **, *p* < 0.01, *n.s.*, not significant, based on one-way ANOVA with Tukey's test. *H*, method for quantification of circular-to-linear telomere DNA ratio in *G*. The signal intensity and area of 2D Southern blotting were measured using the ImageJ software. The *yellow dotted area* (*a*) was used to quantify the circular telomere DNA, and the *magenta dotted area* (*b*) was used to quantify the linear telomere DNA.

Previous studies showed that t-loops lose their stability and are stochastically deleted in cells overexpressing TRF2 ΔB, such that telomeres are rapidly shortened (14). We investigated the change in telomere length by TRF2 RA mutation. TRF2 RA mutation increased telomeric signal free ends at a similar rate to TRF2 ΔB, as assessed by metaphase fluorescence *in situ* hybridization (FISH) using a telomeric TTAGGG probe (Fig. 5, *A* and *B*). In most chromosomes, loss of telomeric signal happened only in one of the sister chromatids in TRF2 RA mutant cells, suggesting that loss of the interaction between TRF2 and the core histones results in deletion of telomeres after telomere replication or in incomplete telomere replication. This finding is compatible with the observation of unprotected telomeres in the S/G_2_ cell cycle phase (Fig. 4, *F* and *G*). We analyzed telomere length by telomeric Southern blotting. The signals of telomeric restriction fragments were rapidly lost in TRF2 RA-expressing cells, similarly to that in TRF2 ΔB-expressing cells (Fig. 5, *C* and *D*). Quantitative analysis of TTAGGG repeat signals showed that 20–30% of telomeric DNA was lost in TRF2 RA mutant-expressing cells, comparable with that in TRF2 ΔB introduced cells.

It has been reported that arginine residues, including the conserved arginines required for histone binding in the GAR domain, are the substrate of protein arginine methyltransferase 1 (PRMT1). The TRF2 RK mutant in which the conserved arginine residues are replaced by lysine in the GAR domain retained its affinity for the core histones (Fig. 2, *E* and *F*) and resulted no significant changes to telomeric DNA (Fig. 5*C*), whereas it induced TIF formation as reported (23) (Fig. 4*D*).

Finally, we studied the stability of the t-loop in TRF2 RA cells. It has been reported that rapid loss of telomeric DNA in TRF2 ΔB-expressing cells is caused by the cleavage of the t-loop and the generation of telomeric DNA with a circular formation (t-circle). We studied the cleavage of t-loops by a t-circle amplification assay (24, 25) and by conventional neutral-neutral two-dimensional (2D) gel electrophoresis (14). Both assays revealed that t-circles accumulated in TRF2 RA cells at a similar rate to those in TRF2 ΔB cells (Fig. 5, *E–H*).

Taken together, these results indicated that loss of the interaction between TRF2 and the core histones decreases the stability of t-loops such that telomeric DNA is rapidly lost.

##### Dimerization of the GAR Domain-deleted TRF2 and Histone H2B Restores Telomere End Protection and t-loop Stability

**FIGURE 6.**
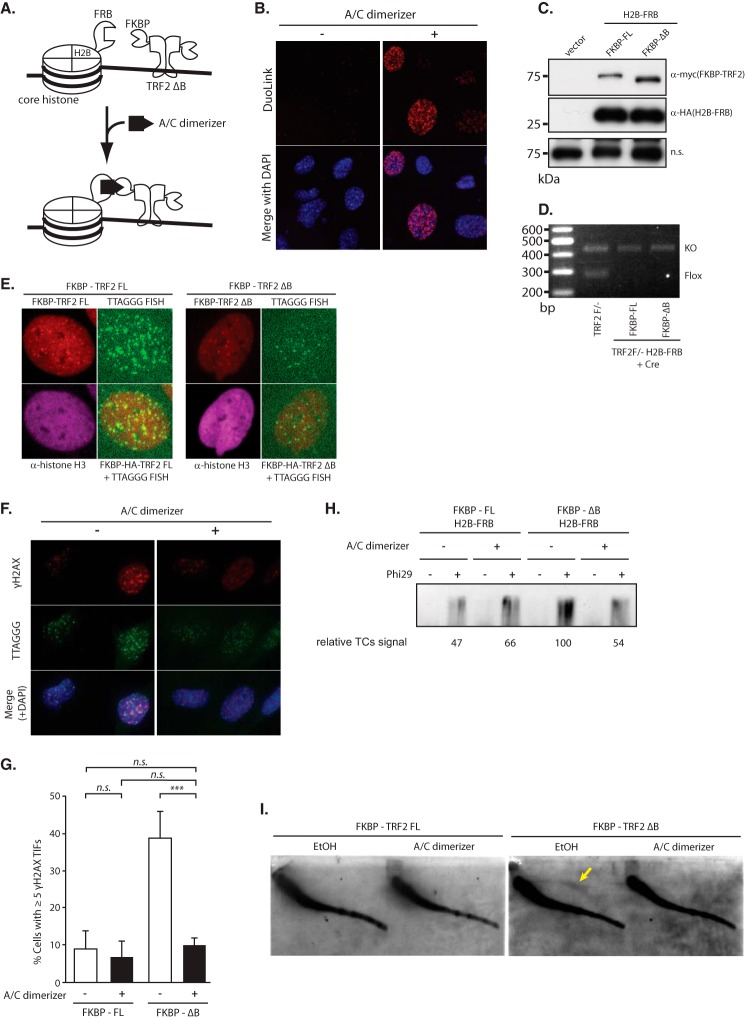
**Regain of telomere protection by forced dimerization of TRF2 and core histone, independent of the GAR domain.** *A*, schematic of the experiment for A/C dimerizer-induced dimerization of FRB-fused histone H2B and FKBP-fused TRF2 ΔB. *B*, Duolink PLA assay to detect the dimerization between histone H2B-FRB and FKBP-TRF2 ΔB. FKBP-TRF2 ΔB-complemented MEFs expressing histone H2B-FRB were incubated with A/C dimerizer for 12 h and then subjected to Duolink PLA assay using antibody against HA (H2B-FRB) and Myc (FKBP-TRF2 ΔB). *C*, immunoblotting to verify the equal expression of Myc-tagged FKBP-TRF2 FL and FKBP-TRF2 ΔB and HA-tagged histone H2B-FRB. Nonspecific (*n.s.*) signal served as a loading control. *D*, PCR to verify Cre-mediated deletion of the endogenous allele from TRF2^F/−^ MEFs expressing H2B-FRB transformed to express FKBP-TRF2 FL and FKBP-TRF2 ΔB. *E*, IF-FISH images to verify equal localization of Myc-tagged FKBP-FL and FKBP-ΔB TRF2 at telomeres. IF-FISH co-staining results using anti-Myc (*red*) and anti-histone H3 (*magenta*) antibodies in conjunction with FISH with a telomeric TTAGGG-specific probe (*green*) are presented. *F*, TIF assay in histone H2B-FRB and FKBP-TRF2 ΔB dimerized cells. FKBP-TRF2 ΔB-complemented MEFs expressing histone H2B-FRB were incubated with A/C dimerizer for 4 h and then subjected to a TIF assay. Immunofluorescence results for γH2AX (*top*), FISH using telomeric TTAGGG probe (middle), and merged images with DAPI (*bottom*) are presented. *G*, quantification of TIF assay in *F*. Bars represent mean values from three independent experiments ± S.D. (*error bars*). ***, *p* < 0.001, *n.s.*, not significant, based on two-way ANOVA with Sidak's test. *H*, inhibition of t-circle production by dimerization of histone H2B and TRF2 ΔB. FKBP-TRF2 ΔB-complemented MEFs expressing histone H2B-FRB were incubated with A/C dimerizer for 48 h and then subjected to t-circle amplification assay. The *numbers* below the lane represent the relative t-circle signal (*TCs*) to A/C dimerizer negative control. *I*, t-circle detection in histone H2B-FRB and FKBP-TRF2 ΔB dimerized cells by 2D Southern blotting. FKBP-TRF2 ΔB-complemented MEFs expressing histone H2B-FRB were harvested 48 h after the addition of A/C dimerizer. Equal amounts of MboI-digested genomic DNA were subjected neutral 2D agarose electrophoresis. Telomeric DNA was visualized by Southern blotting using a DIG-labeled TTAGGG probe. An *arrow* indicates t-circles.

The GAR domain of TRF2 has been reported to be bound directly to a junction DNA or a telomeric repeat containing RNA (15, 18). Methylation of arginine residues in the GAR domain has also been reported to be important to stabilize chromosome ends (23). To investigate the telomere function regulated by the TRF2 and core histone interaction, excluding the complicated regulation of the GAR domain by nucleotide association or post-transcriptional modification, we set up experiments in which the TRF2 ΔB protein was forcibly anchored onto the chromatin. We used the rapamycin-induced heterodimerization system using the FK506-binding protein (FKBP) and FKBP12-rapamycin-binding (FRB) domain of mammalian target of rapamycin (mTOR). The A/C dimerizer, a rapamycin derivative, binds both FKBP and FRB simultaneously, as a result of which FKBP and FRB fusion proteins are forced to dimerize (26). FRB was fused to the C terminus of histone H2B (H2B-FRB), and FKBP was fused to the N terminus of TRF2 ΔB (FKBP-TRF2 ΔB). TRF2 was capable of binding to histone H2B by the A/C dimerizer independently of the GAR domain in the H2B-FRB- and FKBP-TRF2 ΔB-expressing cells (Fig. 6, *A*, *C*, and *D*). FKBP-TRF2 ΔB was located on telomeres, and H2B-FRB was expressed throughout the nuclei (Fig. 6*E*). As expected, FKBP-TRF2 ΔB bound to H2B-FRB in an A/C dimerizer-dependent manner, which was assessed by PLA Duolink assay (Fig. 6*B*).

Mouse embryonic fibroblast (MEF) cells expressing H2B-FRB reconstituted endogenous TRF2 with FKBP-TRF2 ΔB showed TIFs at a similar rate to the cells expressing TRF2 ΔB. Forced dimerization of TRF2 ΔB and histone H2B by A/C dimerizer significantly decreased TIF formation (Fig. 6, *F* and *G*). We also assessed the stability of the t-loop in FKBP-TRF2 ΔB cells with A/C dimerizer. t-circles accumulated in FKBP-TRF2 ΔB cells; however, the generation of t-circles decreased to 50% when the A/C dimerizer was added (Fig. 6, *H* and *I*).

Taken together, these results indicated that the association of TRF2 and the core histones is sufficient to protect the chromosome ends and stabilize the t-loop.

## Discussion

In this study, we unraveled an unexpected connection between TRF2 and core histones. Our data indicated that TRF2 binds directly to core histones through its N terminus GAR domain to protect chromosome ends from DNA damage response and DNA degradation. This interaction requires four arginine residues that are conserved in the GAR domain of mammalian TRF2 proteins (this study) and in Kaposi sarcoma virus protein LANA, which is bound to histone H2A/H2B dimer to anchor viral episome to chromatin in host cells (22). The LANA basic domain docks to the acidic groove on the surface of H2A/H2B dimer. Similar to LANA, the GAR domain of TRF2 also binds to the globular domain of core histones. The TRF2 GAR domain, however, not only binds to H2A/H2B, but also to H3H4, whereas LANA binds only to H2A/H2B. In addition to the three arginine residues of LANA, which are important for histone binding, the TRF2 basic domain contains an extra arginine residue within the binding region. This sequence difference might change the binding specificity of TRF2 basic domain to core histones.

Our data showed that the binding of TRF2 to the core histones is required to stabilize the telomeric loop structure. Loss-of-function mutation in the GAR domain of TRF2 for binding to the core histone induced the rapid telomeric DNA loss and t-circle formation. Holliday junction (HJ) resolution has been reported to be involved in TRF2 ΔBasic-induced rapid telomeric DNA deletion and generation of t-circles (14). In addition, repositioning of the nucleosome is an essential step for HR, and a histone octamer inhibits the branch migration of an HJ that is needed to complete the HR event (27, 28). Nucleosomes at telomere chromatin are considered to have a higher intrinsic mobility than those in the bulk chromatin because telomeric TTAGGG repetitive DNA sequences are not suitable to configure the nucleosomes (9). The interaction between TRF2 and the core histones could increase the stability of the nucleosomes at telomeres and prevent branch migration and resolution of the HJ in the telomeric loop structure.

The GAR domain of TRF2 has been shown to bind to DNA four-way junctions in a structure-specific manner or to the TERRA (15, 16, 18). It is possible that the GAR domain of TRF2 is bound to the core histones with nucleic acid bridging them. Our data, however, indicated that it is unlikely that the interaction between the GAR domain of TRF2 and the core histones is dependent on RNA or DNA because RNase A or DNase I treatment had a very limited effect on the *in vitro* binding of the GAR domain of TRF2 to the core histones (Fig. 2*A*). It is still difficult to exclude the possibility that the function of the GAR domain of TRF2 relies on the nucleic acid interaction. Chemically induced dimerization of TRF2 ΔBasic and histone H2B, however, could rescue the GAR domain deletion phenotype, such as DNA damage response at telomeres or t-circle formation (Fig. 6, *F–I*). This experiment showed that the association of TRF2 and the core histones is sufficient to protect the chromosome ends and stabilize the t-loop because the nucleic acid is not required for the chemically induced binding of TRF2 and core histones in this assay. This result, of course, cannot exclude the possibility that the nucleic acid interaction of TRF2 contributes to the telomere protection.

The GAR domain of TRF2 was also reported as a substrate of PRMT1, and the methylation of arginine residues in the GAR domain is involved in the regulation of telomere function. The TRF2 RK mutant, which has arginine-to-lysine substitutions in the GAR domain that do not change the positive charge of the basic domain, is not recognized as a substrate by PRMT1. Overexpression of TRF2 RK induced TIF formation and cellular senescence similar to TRF2 ΔBasic (23). TRF2 basic RK mutant was still bound to the core histone (Fig. 2*F*). This seems to contradict our results indicating that the interaction between TRF2 and core histone is required for telomere protection. However, the consequence of TRF2 RK mutant on t-loop stability is completely different from TRF2 ΔBasic and TRF2 RA mutant. Both TRF2 ΔBasic and TRF2 RA mutants induce rapid telomeric DNA loss and t-circle formation, whereas the TRF2 RK mutant does not (23) (Fig. 5*C*). The molecular mechanism of telomere protection by TRF2 is complicated, such that the regulation of telomere function by TRF2 methylation in the GAR domain might differ from the TRF2-core histone interaction. The chemically induced dimerization of TRF2 ΔBasic and histone H2B experiments support this hypothesis.

The shelterin complex regulates the telomeric chromatin structure. Overexpression of TRF2 induces an aberrant nucleosomal structure (29) and changes histone post-transcriptional modification at telomeres (30). The acidic groove in the surface of histone H2A/H2B dimer where the LANA basic domain binds is the same region where the histone H4 N-tail binds (31). The interaction between the histone H4 N-tail and histone H2A/H2B dimer acidic patch has been proposed to be important for the higher order chromatin structure (32, 33). Recently, it has been reported that LANA recruits the histone demethylase, KDM3A, and regulates the virus epigenome chromatin (34). Thus, the GAR domain of TRF2 could potentially regulate the higher order chromatin structure at telomeres through the association with the globular domain of core histones.

## Experimental Procedures

### 

#### 

##### Cell Culture

HeLa cells, IMR90 cells, NIH-3T3 cells, and MEF cells were maintained in DMEM (Nacalai Tesque, Kyoto, Japan) containing 10% FBS, minimum essential medium non-essential amino acid, l-glutamine, and penicillin-streptomycin (GIBCO^TM^, Thermo Fisher). TRF2 ts and TRF2 ΔB ts cells were maintained at 32 °C.

##### Antibodies and Reagents

An antibody against hTRF2 was purchased from IMGENEX (San Diego, CA). Anti-histone H3, anti-GFP, and anti-HA antibodies were from Abcam (Cambridge, UK), and anti-GAPDH, anti-cyclin A, anti-GFP, and anti-Myc antibodies were purchased from Santa Cruz Biotechnology (Santa Cruz, CA). Anti-H2B and anti-γ H2AX antibodies were purchased from Millipore (Billerica, MA). Anti-histone H3K9 methylation-, H3K27 tri-methylation-, and H4K20 mono-methylation-specific antibodies were a kind gift from David Allis. The Duolink PLA assay kit was purchased from Sigma-Aldrich. The A/C dimerizer was purchased from Clontech.

##### Plasmids

The N-terminal Myc-tagged mouse TRF2 FL, TRF2ΔBasic (aa 45–497), TRF2ts (TRF2 I468A), and TRF2ts ΔBasic expression plasmids were constructed by cloning cDNA for mTRF2 into the pWzl plasmid (12, 19). The N-terminal Myc-tagged human TRF2 FL and TRF2ΔBasic (aa 45–500) expression plasmids were constructed by cloning cDNA for hTRF2 into pLPC (35). Alanine or lysine mutations of TRF2 basic were prepared by PCR-mediated mutagenesis using a QuikChange Lightning site-directed mutagenesis kit (Stratagene, La Jolla, CA), according to the manufacturer's instructions. cDNA encoding the GST fusion hTRF2 basic domain was cloned into plasmid pGEX (Amersham Biosciences). The C terminus NLS-EGFP fusion TRF2 basic domain expression plasmid was constructed by cloning mTRF2 basic domain (aa 2–51) and 3× tandem repeats of the SV40 NLS into plasmid pMSCV-EGFP (36). The histone H2B-FRB expression plasmid was generated by cloning mouse H2B without a stop codon and FRB from Lyn-linker-FRB (Addgene plasmid 20147 (26)) into pMSCV vector. FKBP-TRF2 and FKBP-TRF2 ΔB plasmids were constructed by cloning FKBP from YFP-FKBP-Rac1CA (Addgene plasmid 20150 (26)) into vectors pLPC-TRF2 and pLPC-TRF2 ΔB.

##### Retroviral Vector Production and Infection

Plat-E retroviral packaging cells (37) were transfected with the desired DNA plasmids. At 48, 60, and 72 h after transfection, the viral medium was collected and used to infect cells in the presence of Polybrene (4 μg/ml). At 48 h after the last infection, cells were selected with the desired drug and then maintained in selection medium.

##### Image Analysis

An inverted microscope (Olympus IX71) equipped with a cooled CCD camera (Olympus DP71) using 40× (0.85 NA) and 60× (1.2 NA) objectives (Olympus, Tokyo, Japan) or an IN Cell Analyzer 1000 equipped with a 20× objective (GE Healthcare Life Science) was used to acquire the fluorescence images. The images were processed using Photoshop (Adobe, San Jose, CA).

##### Statistical Analyses

Statistical analyses were performed by unpaired Student's *t* test, one-way ANOVA with Dunnett's test, one-way ANOVA with Tukey's test, or two-way ANOVA with Sidak's test using the GraphPad Prism software (GraphPad Software Inc., La Jolla, CA).

##### TIF Assay

Immunofluorescence-fluorescent *in situ* hybridization (IF-FISH) was used to detect TIFs as described previously (19), using primary antibodies against γH2AX. Briefly, cells grown on coverslips were fixed for 15 min in 2% paraformaldehyde at room temperature, followed by 15 min in 100% methanol at −20 °C. After rehydration in PBS for 5 min, cells were incubated for 30 min in Blocking Solution (1 mg/ml BSA, 3% goat serum, 0.1% Triton X-100, 1 mm EDTA in PBS). The cells were incubated with primary antibodies in Blocking Solution for 1 h at room temperature, washed three times in PBS, incubated with secondary antibodies in Blocking Solution for 30 min, and washed again three times in PBS. At this point, coverslips were fixed with 2% paraformaldehyde for 15 min at room temperature, washed twice in PBS, dehydrated consecutively in 70, 95, and 100% ethanol for 5 min each, and allowed to dry completely. Hybridizing Solution (70% formamide, 1 mg/ml blocking reagent (Roche Applied Science), 10 mm Tris-HCl, pH 7.2, containing peptide nucleic acid (PNA) probe FITC-OO-(CCCTAA)3 (Bio-Synthesis, Lewisville, TX)) was added to each coverslip, and the cells were denatured by heating for 10 min at 80 °C on a heat block. After 2 h of incubation at room temperature in the dark, the cells were washed twice with Wash Solution (70% formamide, 10 mm Tris-HCl, pH 7.2) and twice in PBS. Slides were mounted in ProLong Gold antifade reagent with DAPI (Invitrogen). Cells were blindly scored for five or more telomeric γH2AX foci in three independent experiments. At least 250 cells were scored in each experiment.

##### Telomere FISH on Metaphase Spreads

Telomere FISH was performed as described previously (19). Cells were harvested at the indicated time points and fixed. Metaphase spreads were aged overnight, and PNA FISH was performed. Slides were washed in PBS once and dehydrated by consecutive 5-min incubations in 70, 95, and 100% ethanol. After air-drying, hybridizing solution (as in IF-FISH) containing the FITC-OO-(AATCCC)3 PNA probe (Bio-Synthesis) was added, and spreads were denatured by heating for 10 min at 80 °C on a heat block. Spreads were hybridized in the dark for 2 h at room temperature. Two 15-min washes were performed in 70% formamide, 10 mm Tris-HCl, pH 7.0, 0.1% BSA, followed by three washes in 0.1 m Tris-HCl, pH 7.0, 0.15 m NaCl, 0.08% Tween 20 with DAPI added to the second wash to counterstain the chromosomal DNA.

##### Pulsed-field Gel Electrophoresis and Detection of Telomeric DNA

Contour-clamped homogenous electric field (CHEF) gel electrophoresis of mouse DNA was performed essentially as described previously (12). Cells were resuspended in PBS and mixed 1:1 (v/v) with 2% agarose to obtain 1 × 10^6^ cells per agarose plug, digested in proteinase K buffer (100 mm EDTA, pH 8.0, 0.2% sodium deoxycholate, 1% sodium lauryl sarcosine, 1 mg/ml proteinase K) and washed extensively in TE buffer (10 mm Tris, pH 8, 1 mm EDTA). Plugs were incubated overnight at 37 °C with 60 units of MboI in 0.5 ml. Following digestion, plugs were washed in TE and equilibrated in 0.5× TBE before loading into a 1% agarose-0.5× TBE gel. The gel was run using CHEF-DR II apparatus (Bio-Rad) in 0.5× TBE for 24 h with the following settings: initial pulse, 5 min; final pulse, 5 min; 6 V cm^−1^ at 14 °C, and then transferred to Hybond-N membrane. The blots were first hybridized with DIG-labeled a TTAGGG repeat probe, and then stripped with 0.1× SSC and 0.2 n NaOH at 37 °C and probed with a DIG-labeled major satellite probe. The following oligonucleotide probes were used for detection: major satellite (60-mer GenBank^TM^ accession number X06899, base pair 1–60) (38) and telomeric probe (24-mer, 5′-(TTAGGG)×4-3′). Relative telomere repeat signals were quantified from at least three independent experiments. Signal increase represented the percentage of increase of average telomere signals normalized to its major satellite control and relative to the wild-type (wt) TRF2 control.

##### Purification of Core Histones from HeLa Cell Nuclei and Generation of Tailless Core Histones

Core histones and nucleosome were prepared from the HeLa cells as described previously (39). Briefly, mono-nucleosomes were purified using a hydroxyapatite column from HeLa cell nuclei digested with micrococcal nuclease. The nucleosome fractions were eluted with sodium phosphate and analyzed by agarose gel electrophoresis. Clean mono-nucleosome peaks were collected and partially digested with trypsin to generate the tailless histones. Core histones were purified with a hydroxyapatite column from sonicated HeLa cell nuclei. The column was washed with 0.63 m NaCl and then eluted with 2 m NaCl for core histones. Histone H2A/H2B and H3/H4 were eluted with 1 m and 2 m NaCl from hydroxyapatite column respectively.

##### In Vitro Release of TRF2ts Protein from Nuclei

Cells were washed in cold PBS, resuspended in Solution A (10 mm Hepes, pH 7.9, 10 mm KCl, 1.5 mm MgCl_2_, 0.34 m sucrose, 10% glycerol, 0.1% Triton X-100, 1 mm DTT, 1 mm PMSF, protease inhibitor cocktail), incubated on ice for 10 min, and centrifuged at 1,300 × *g* for 4 min. Nuclei collected in pellet 1 (P1) were washed with Solution A twice, lysed in Solution B (3 mm EDTA, 0.2 mm EGTA, 1 mm DTT, protease inhibitor cocktail) on ice for 30 min, centrifuged at 1,700 × *g* for 4 min, washed twice in Solution B, and centrifuged again under the same conditions. Chromatin-bound fraction was collected in pellet 2 (P2). P2 was resuspended in a small volume of Solution B, incubated at 37 °C for 30 min, and centrifuged. *In vitro* chromatin-released fraction was collected in the supernatant and used for co-immunoprecipitation experiments. For pretreatment of *in vitro* chromatin-released fraction with DNase I and RNase A, P2 was resuspended in a small volume of Solution C (50 mm Tris-HCl, pH 7.4, 100 mm NaCl, 1% Triton X-100, 1 mm PMSF, and Complete protease inhibitor), incubated at 37 °C for 30 min, and centrifuged. 2.5 mm MgCl_2_ and 0.5 mm CaCl_2_ were added to the supernatants, and the supernatants were incubated with 100 μg/ml DNase I and 100 μg/ml RNase A at 4 °C for 1 h and then used for co-immunoprecipitation experiments.

##### Co-immunoprecipitation

*In vitro* chromatin-released fraction prepared from the isolated chromatin of TRF2ts MEFs was diluted with IP buffer (20 mm Tris-HCl, pH 7.4, 0.5% Nonidet P-40, 150 mm KCl, 1 mm MgCl_2_, 0.5 mm EDTA, and protease inhibitor cocktail) and incubated with 1 μg of anti-Myc monoclonal antibody (9E10, Santa Cruz Biotechnology) for 2 h at 4 °C. Immunoprecipitates were prepared by incubation with Dynabeads protein G (Invitrogen) and subjected to immunoblotting with the indicated antibodies. Cell extracts prepared from NIH-3T3 cells that were transfected with Basic-NLS-EGFP, Basic-RA-NLS-EGFP, and NLS-EGFP expression plasmids using radioimmunoprecipitation assay buffer without SDS (Nacalai Tesque) were incubated with 1 μl of anti-GFP polyclonal antibody (Abcam) for 2 h at 4 °C. Immunoprecipitates were prepared by incubation with Dynabeads protein A (Invitrogen) and subjected to silver staining and immunoblotting with the indicated antibodies. Silver staining was performed using the Sil-Best Stain One kit (Nacalai Tesque), according to the manufacturer's instructions.

##### In Vitro Binding Assay

GST fusion proteins were produced in BL21-AI *Escherichia coli* strain (Invitrogen) by isopropyl β-d-1-thiogalactopyranoside induction, extracted in extraction buffer (50 mm Tris-HCl, pH 7.4, 100 mm NaCl, 1 mg/ml lysozyme, 1 mm MgCl_2_, 1% sodium lauryl sarcosine, 1% Triton X-100, 1 mm PMSF, Complete protease inhibitor). To remove DNA or RNA from the *in vitro* binding assay, the bacterial extracts and purified core histone were treated with 100 μg/ml DNase I and 100 μg/ml RNase A at 4 °C for 1 h in DNase I buffer (50 mm Tris-HCl, pH 7.4, 100 mm NaCl, 2.5 mm MgCl_2_, 0.5 mm CaCl_2_, and Complete protease inhibitor). GST fusion proteins were then captured by glutathione-Sepharose beads (GE healthcare). Beads were washed with high-salt IP buffer (20 mm Tris-HCl, pH 7.4, 0.5% Nonidet P-40, 1 m KCl, 1 mm MgCl_2_, 0.5 mm EDTA, and protease inhibitor cocktail) twice and IP buffer (20 mm Tris-HCl, pH 7.4, 0.5% Nonidet P-40, 150 mm KCl, 1 mm MgCl_2_, 0.5 mm EDTA, and protease inhibitor cocktail) twice. Beads were then incubated with core histones in IP buffer containing 50 μg/ml ethidium bromide at 4 °C for 2 h, washed with IP buffer extensively, resuspended in 2× Laemmli buffer, and subjected to Coomassie Brilliant Blue (CBB) staining and immunoblotting with the indicated antibodies. CBB staining was performed using the Quick-CBB kit (Wako Pure Chemical Industries, Osaka, Japan), according to the manufacturer's instructions.

##### Telomeric Circle Assay

Detection of telomeric circles was performed essentially as described previously (24, 25). Briefly, extracted genomic DNA was double-digested by AluI/HinfI restriction enzymes overnight, precipitated, and used for the telomeric circle assay. Digested genomic DNA was annealed with mammalian telomere primer (5′-CCCTAACCCTAACCCTAAccc-3′; the three terminal 3′-nucleotides indicated with small letters were synthesized using thiophosphate linkages), amplified with Phi29 DNA polymerase (Fermentas, Vilnius, Lithuania), subjected to alkaline gel electrophoresis, and transferred to a Hybond-N membrane. The blots were hybridized with a DIG-labeled TTAGGG repeat probe.

##### Pre-extraction of the Nucleoplasmic Fraction

Pre-extraction to remove the nucleoplasm was performed as described previously (40). Briefly, cells were seeded onto glass coverslips. The coverslips were washed in cold PBS and incubated in cytoskeleton buffer (10 mm PIPES (pH 6.8), 100 mm NaCl, 300 mm sucrose, 3 mm MgCl_2_, 1 mm EGTA, 0.5% Triton X-100) for 5 min on ice, followed by incubation in cytoskeleton stripping buffer (10 mm Tris-HCl (pH 7.4), 10 mm NaCl, 3 mm MgCl_2_, 1% Tween 40, 0.5% sodium deoxycholate) for 5 min on ice. After several washes with ice-cold PBS, the cells were fixed in 2% paraformaldehyde for 15 min.

##### Duolink PLA Assay

The Duolink PLA assay was performed according to the manufacturer's instructions. Briefly, cells were grown on coverslips and then fixed in 2% paraformaldehyde for 15 min before permeabilization in PBS containing 0.5% Nonidet P-40 for 15 min. Cells were then blocked by incubation for 30 min in PBS with 0.2% cold-water fish gelatin and 0.5% BSA and incubated with primary antibodies. After washing the cells, PLA probes were added, followed by hybridization, ligation, and amplification at 37 °C. DNA was stained with DAPI. Images were analyzed by the IN Cell Analyzer 1000 software. Cells were scored for 10 or more Duolink signals in nuclei in three independent experiments. At least 250 cells were scored in each experiment.

##### SA-β-gal Assay

The SA-β-gal assay was performed with a Senescence Detection Kit (Abcam) according to the manufacturer's instructions.

## Author Contributions

S. S. and T. I. analyzed the data and critiqued the paper. A. K. designed and performed the experiments, analyzed the data, and wrote the manuscript.

## Footnotes

2 The abbreviations used are:

HR: homologous recombination
TRF2: telomeric repeat-binding factor2
TRF2ts: temperature-sensitive TRF2 mutant
TIF: telomere dysfunction-induced foci
GAR domain: Gly/Arg-rich domain
TERRA: telomere repeat-encoding RNA
PLA: proximity ligation assay
FL: full-length
LANA: latency-associated nuclear antigen
NLS: nuclear localization signal
EGFP: enhanced green fluorescent protein
PRMT1: protein arginine methyltransferase 1
SA-β-gal: senescence-associated β-galactosidase
FKBP: FK506-binding protein
FRB: FKBP12-rapamycin-binding
MEF: mouse embryonic fibroblast
HJ: Holliday junction
aa: amino acids
ANOVA: analysis of variance
IF-FISH: immunofluorescence-fluorescent *in situ* hybridization
TBE: Tris-borate-EDTA
PNA: peptide nucleic acid
DIG: digoxigenin
CBB: Coomassie Brilliant Blue
CHEF: contour-clamped homogenous electric field
IP: immunoprecipitation
h: human
m: mouse.
